# Coronary Cannulation Following Transcatheter Aortic Valve Replacement With Self-Expanding Evolut FX+ System: The CANNULATE TAVR II Study

**DOI:** 10.1016/j.shj.2025.100483

**Published:** 2025-04-23

**Authors:** Rafey Feroze, Marco Frazzetto, Lakshmi Prasad Dasi, Taylor Becker, Luis Augusto Palma Dallan, Anene Ukaigwe, Steven J. Filby, Gregory Rushing, Guilherme F. Attizzani

**Affiliations:** a Division of Cardiology, Harrington Heart and Vascular Institute, University Hospitals Cleveland Medical Center, Cleveland, Ohio, USA; b Department of Biomedical Engineering, Georgia Institute of Technology, Atlanta, Georgia, USA; c Department of Biomedical Sciences, Ohio State University, Columbus, Ohio, USA; d Division of Cardiovascular Surgery, Harrington Heart and Vascular Institute, University Hospitals Cleveland Medical Center, Cleveland, Ohio, USA; e Department of Cardiology, Osaka University, Osaka, Japan

**Keywords:** Cannulation, Evolut FX+, TAVR

## Abstract

• The CANNULATE TAVR II is the first prospective study evaluating coronary cannulation following implantation of the self-expanding Evolut FX+ valve, offering a direct comparison with patients treated using the previous-generation Evolut FX platform.
• A novel geometric analysis using preprocedural and postprocedural computed tomography imaging was introduced to assess the spatial relationship between valve apertures and coronary ostia, incorporating the left coronary artery-right coronary artery angle and coronary height differences.
• Mean time for the cannulation of the right coronary artery was faster with the newer Evolut FX+ platform compared to the previous generation, despite an experienced operator performing the procedures.
• Despite approximately half of the coronaries not being within the apertures of the newer Evolut FX+ platform after post-transcatheter aortic valve replacement computed tomography-scan analysis, a successful coronary cannulation was obtained in 100% of the cases using the same approach as the prior generation, and no modifications were implemented to ensure that the apertures were co-axial with the coronaries.

Coronary cannulation (CC) after transcatheter aortic valve replacement (TAVR) is crucial as the indication expands to younger patients.^1^ The Evolut FX valve (Medtronic Inc, Minneapolis, Minnesota) allows fluoroscopic assessment of commissure alignment by the golden markers positioned at its inflow and commissural alignment has been associated with improvement in coronary access following valve implantation.^2^^,^^3^^,^^4^ The newer Evolut FX+ (Medtronic Inc, Minneapolis, Minnesota) has 3 apertures positioned 120 degrees apart designed to facilitate CC. The aim of our prospective study is to describe the rate of successful CC following the Evolut FX+ implantation, comparing it with a cohort of Evolut FX while performing a computed tomography (CT) analysis of the relationship of the apertures and coronary ostia.

We included patients who underwent TAVR with Evolut FX+ and compared CC with a cohort of patients treated with Evolut FX.^2^ Preprocedural CT imaging was analyzed as previously described.^5^ Additionally, the left coronary artery (LCA) to right coronary artery (RCA) angle was measured in a short axis (axial) view. The TAVR procedure was performed and CC was performed immediately afterward as previously described.^2^^,^^5^ Post-TAVR CT imaging was used to obtain the distance of the coronary arteries from the inflow of the valve and the angle of the coronary artery ostium to the center of the closest aperture. The geometric possibility of ideal Evolut FX+ implantation to allow for alignment of the coronary arteries with the apertures was assessed using the formula in Figure 1a to obtain the required left main coronary artery-RCA angle range for each of the four sizes of the Evolut FX+, while accounting for the height difference between the coronaries. The protocol was approved by the institutional review board, and informed consent was obtained.

**Figure 1:**
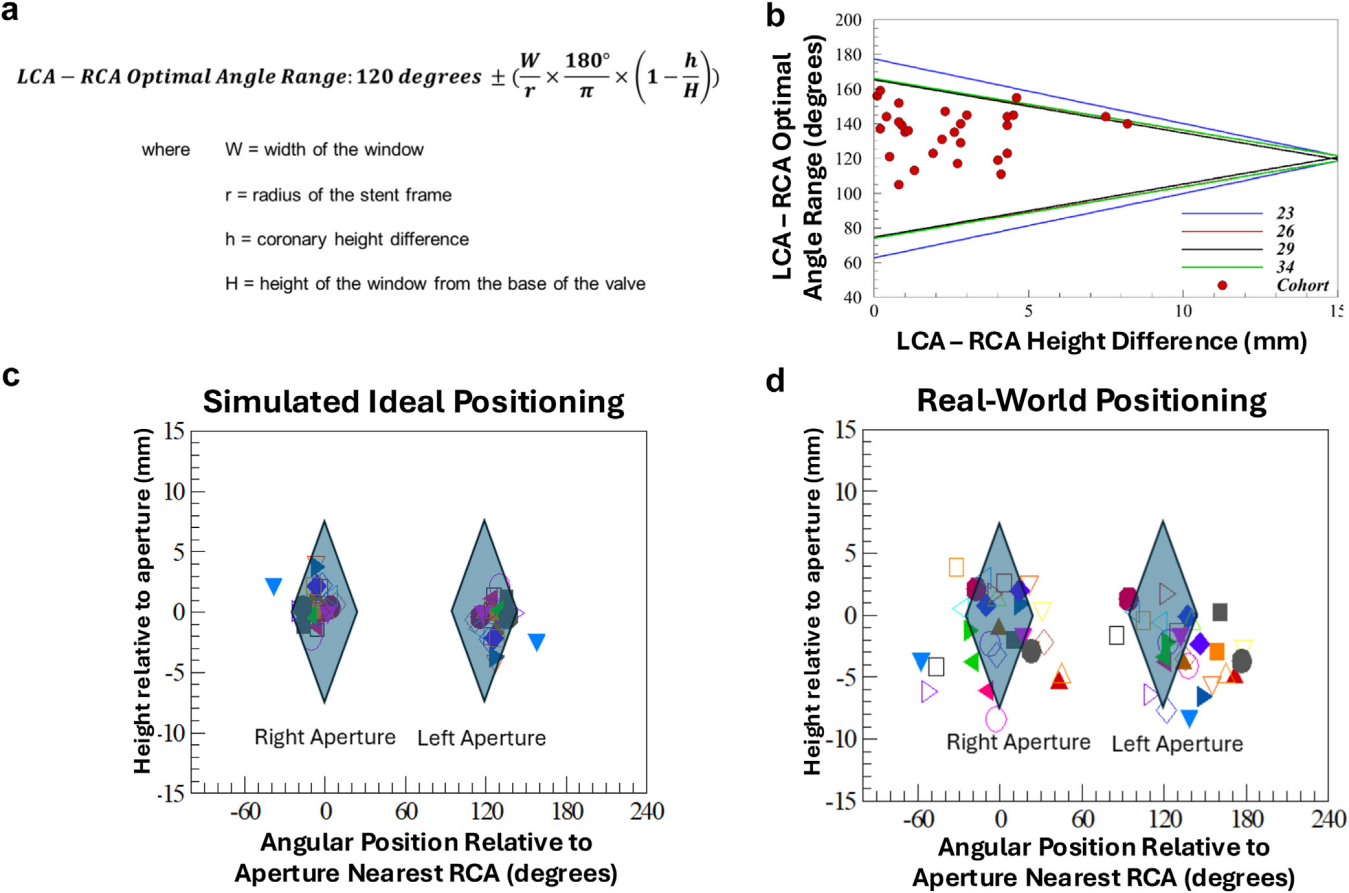
Panel (a): Mathematic equation for predicting the possibility of alignment between the apertures and coronary ostia. Panel (b): Predicted possibility of alignment between apertures and coronary ostia based on LCA-RCA angle ranges and LCA-RCA height difference for the four valve sizes based on the Evolut FX+ cohort. Panel (c): Simulation-based possibility of alignment between left and right apertures with left main and right coronary artery in the Evolut FX+ population. Panel (d): Final postprocedural position and alignment of the apertures and coronary ostia. Abbreviations: LCA, left coronary artery; RCA, right coronary artery.

Our cohort included 128 patients, with 34 consecutive patients receiving the Evolut FX+ and 94 patients receiving the Evolut FX. Fluoroscopic commissure alignment was achieved for all patients, and no patients required recapture and rotation of the delivery system due to misalignment. CC was achieved in 100% of the patients in both groups. There were no significant differences in selective cannulation of the LCA (Evolut FX: 70.2% vs. Evolut FX+: 67.6%, *p* = 0.781) and RCA (Evolut FX: 63.8% vs. Evolut FX+: 67.9%, *p* = 0.689). Mean time for LCA cannulation was similar between the two valves (Evolut FX: 113 ± 9 vs. Evolut FX+: 100 ± 13 seconds, *p* = 0.242), while RCA cannulation was faster with the newer generation valve (Evolut FX: 149 ± 13 vs. Evolut FX+: 100 ± 14 seconds, *p* = 0.003).

Post-TAVR CTs were available for 30 Evolut FX+ patients. The mean annulus perimeter was 75.87 ± 5.87 mm and the mean LCA-RCA angle was 135.3 ± 14.0 degrees. The mean angle between the center of the closest aperture and the LCA ostium was 21.5 ± 17.1 degrees, and for the RCA ostium, it was 21.9 ± 17.1 degrees. In 93.3% (28/30) and 96.7% (29/30) of cases, the LCA and RCA, respectively, were positioned vertically (long axis of the valve) at the level of the apertures. Furthermore, 53.3% (16/30) of LCAs and 43.3% (13/30) of RCAs were within the axial range of the closest aperture.

The required LCA-RCA angle ranges for the four valve sizes that would allow the coronaries to be within the large windows (in the setting of optimal Evolut FX+ alignment) are illustrated graphically in Figure 1b (across coronary height differences of 0-14 mm). As expected, the required LCA-RCA angle range decreases with increasing coronary height differences. Notably, 90% (27/30) of patients’ coronary anatomy was such that it was geometrically feasible for both coronary ostia to be completely within adjacent apertures as illustrated in Figure 1b and c. The remaining three had coronary height differences of >14 mm. The comparison with our real-life results is illustrated in Figure 1d.

The present study is the first experience with CC following Evolut FX+ implantation. Similar to the CANNULATE TAVR study with the Evolut FX, there was 100% successful CC in the present cohort.^2^ While there was no difference in CC success across both valves, 95% of the comparator group (i.e., 94 patients from the Evolut FX cohort) was cannulated by the same experienced operator (G.F.A.), which may have enhanced the performance of that group. Despite the operator’s experience, we observed shorter cannulation time for the RCA and LCA (when the LCA ostia were at the level of apertures in the long axis of the valve) with the new Evolut FX+ platform, which may be related to easier catheter manipulation within the apertures.

As Figure 1d demonstrates, approximately half of the coronaries were not within the apertures. Importantly, our valves were implanted using the same approach as the prior generation, and no modifications were implemented to ensure that the apertures were co-axial with the coronaries. Our LCA-RCA angle range analysis (Figure 1a-c) collectively confirms the geometric possibility for valve implantation that would allow both coronary ostia to be within an aperture in the majority of cases, particularly when the LCA-RCA angle is between 100 and 150 degrees, and the coronary height difference is ≤ 5 mm. Future studies utilizing patient-specific information from the pre-TAVR CT will be required to customize Evolut FX+ implantation depth and valve rotation to optimize aperture location and subsequent CC.

## Ethics Statement

This study was conducted in accordance with the ethical standards of our institutional research committee and with the 1964 Helsinki Declaration.

## Funding

The authors have no funding to report.

## Disclosure Statement

R. Feroze is a consultant for Beckmann Coulter. G. F. Attizzani is a consultant, serves on the advisory board, and has research grants for Medtronic, Boston Scientific, Dasi Simulations, and Elixir. S. J. Filby is a consultant for Boston Scientific. A. Ukaigwe is a consultant for Medtronic. The other authors had no conflicts to declare.
